# Surface Modification and Pore Size Regulation of MSN as Function Aflibercept Carrier for Anti-Vascular Migration

**DOI:** 10.3390/ma18184384

**Published:** 2025-09-19

**Authors:** Ruiqi Guo, Xue Zhang, Yakai Song, Jiachen Shen, Kai Li, Yi Zheng

**Affiliations:** 1 Faculty of Life Science and Medicine, Harbin Institute of Technology, Harbin 150080, China; sheniiachengo@163.com (R.G.); xuezhangm@163.com (X.Z.); 15895433496@163.com (Y.S.); 2 Eye Hospital, The First Affiliated Hospital, Harbin Medical University, Harbin 150001, China; shenjiachengo@163.com

**Keywords:** mesoporous silica, choroidal neovascularization, long-acting therapeutic effect, VEGF, age-related macular degeneration

## Abstract

Age-related macular degeneration (AMD) represents a leading cause of irreversible blindness in the elderly, primarily by choroidal neovascularization (CNV) leakage. While intravitreal injections of anti-angiogenic antibodies (e.g., aflibercept) provide clinical benefits, their short half-life necessitates frequent administrations, potentially causing ocular infections or retinal detachment. There is an urgent need for effective antibody delivery systems. Mesoporous silica nanoparticles (MSN) have emerged as promising nanocarriers due to their tunable porosity, surface modifiability, and biocompatibility, though their application in ophthalmology for antibody delivery remains underexplored. We developed two MSN carries: spiky mesoporous silica nanospheres (S-MSN) without amino groups and amine-functionalized hollow dendritic mesoporous silica nanospheres (A-HDMSN). Characterization revealed that A-HDMSN exhibited superior properties, including a larger surface area (550.32 vs. 257.72 m^2^/g), larger mesoporous pore size (17 vs. <10 nm), and 5.28 times higher drug loading capacity (286.31 ± 8.14 vs. 54.26 ± 3.61 μg/mg) compared to S-MSN (*n* = 3, *p* < 0.001), attributable to pore size effects and hydrogen bonding. FITC-labeled A-HDMSN demonstrated efficient uptake by retinal pigment epithelial cells (ARPE-19). Notably, A-HDMSN loaded with Aflibercept (A-HDMSN@Afl) showed significant inhibitory effect on VEGF-induced cell migration even 10 days after drug release in vitro, indicating a favorable sustained-release effect of the drug. These findings highlight A-HDMSN as a promising antibody delivery platform that could extend clinical dosing intervals, offering potential for improved AMD management.

## 1. Introduction

Age-related macular degeneration (AMD) is one of the leading causes of blindness in the elderly [1,2]. It is estimated that by 2024, the number of AMD patients will reach 288 million cases [3]. AMD has a multifactorial pathogenesis [4,5]. AMD is classified into dry macular degeneration and wet macular degeneration based on different symptoms [6,7]. The main characteristic of dry macular degeneration is atrophy, which is primarily observed. In contrast, wet AMD is characterized by choroidal neovascularization (CNV) and associated leakage [8,9]. The primary clinical treatment methods are intravitreal injections of anti-angiogenic drugs, such as Aflibercept (Afl) and Ranibizumab (Ran) [10,11,12,13]. Afl, a recombinant human Fc-fusion protein, binds to vascular endothelial growth factor A (VEGF-A), vascular endothelial growth factor B (VEGF-B), and placental growth factor (PIGF) [14]. Ran is a humanized antigen-binding fragment of a murine full-length monoclonal antibody directed against human VEGF-A [15]. The half-life of Afl (1.2 mg) and Ran (0.5 mg) in vitreous of rabbits are 3.92 [16] and 2.88 [17] days by intravitreal injection, respectively [18,19,20,21]. This necessitates frequent intravitreal injections for patients. Frequent and multiple drug administrations can cause damage to the normal ocular structure, which may lead to intraocular inflammation, bleeding, and retinal detachment [22]. Therefore, it is necessary to develop a new treatment plan to prolong drug residence time in posterior segment of the eye. Meanwhile, reducing the dosing frequency and extending the dosing interval has the benefit of reducing the incidence of potential thrombosis in patients [23].

Mesoporous silica nanoparticles (MSN) have been developed as an efficient and nontoxic drug delivery system [24,25,26]. In addition, MSN have many advantages, such as tunable particle size, feasible surface modification, three-dimensional (3D) structure [27,28], high porosity [29,30], mature technology [31], easy surface modification [32], and a stable rigid framework [33]. MSN have been explored in many fields [28,34,35]. Studies have shown that polyethylenimine-functionalized hybrid silica spheres exhibit excellent delivery efficiency of siRNA into osteosarcoma cancer cells and human colon cancer cells, resulting in significant cell inhibition [36]. Double-shelled dendritic mesoporous organosilica hollow spheres serve as an excellent adjuvant and provide superior immunity in cancer immunotherapy [28]. Amine-functionalized hollow dendritic mesoporous silica nanospheres enhance the cellular uptake of the hydrolytic β-galactosidase in Chinese hamster ovary cells (CHO-K1) and retain its intracellular catalytic ability, suggesting great potential for intracellular delivery of large biomolecules [37]. Nanoporous silica materials enhance the dissolution of the poorly soluble drugs [29]. The elimination of nanoparticles in the vitreous body is mainly related to their particle size [38]. After intravitreal administration, MSN were eliminated gradually into anterior and posterior routes [39]. The literature reports that the residence time of nanoparticles with a particle size of 200 nm in the vitreous body is 65 days [39]. The main clearance pathways of MSNs from the body include excretion by the kidneys and clearance by the liver and spleen [40]. Despite extensive research on silica nanomaterials in drug delivery, few studies have systematically investigated the relationship between pore architecture and drug loading efficiency. Furthermore, the synergistic effects of hydrogen bonding interactions on drug release remain underexplored. Which significantly limits the rational design of optimized nano-carriers.

Herein, we prepared amine-functionalized hollow dendritic mesoporous silica nanospheres (A-HDMSN) and spiny mesoporous silica nanospheres (S-MSN). The A-HDMSN become suitable drug delivery carriers for their appropriate pore volume and high specific surface area. Surprisingly, A-HDMSN-loaded Aflibercept (Afl) show a long-term effect on VEGF-induced cell migration. Per our analysis, this was due to the combined effect of pore size and hydrogen bonds, which restricted the rapid release of Afl. A-HDMSN demonstrates significant potential as a silicon-based nanocarrier for ocular drug delivery. The optimized pore structure and surface chemistry address key challenges in ophthalmic medication transport. This system shows promise for Afl delivery, with advantages over conventional materials.

## 2. Materials and Methods

### 2.1. Chemicals and Reagent

Hydroquinone (C_6_H_6_O_2_, 99%), formaldehyde (CH_2_O, 37 wt%), ammonia solution (NH_3_∙H_2_O, 28 wt%), triethylamine (TEA, C_6_H_15_N, ≥99%), hexadecyltrimethylammonium chloride (CTAC, C_19_H_42_ClN, 98%), chlorobenzene (C_6_H_5_Cl, 99.9%), (3-Aminopropyl) triethoxysilane (APTES, C_9_H_23_NO_3_Si, 98%), tetraethyl orthosilicate (TEOS, C_8_H_20_O_4_Si, 99.0%), fluorescein isothiocyanate (FITC, C_21_H_11_N_5_S, 90%), and methylthiazolyldiphenyl-tetrazolium bromide (MTT, C_18_H_16_BrN_5_S, 98%) were purchased from Sigma-Aldrich (St. Louis, MI, USA). Methanol (CH_4_O, 99.5%) and ethanol (C_2_H_5_OH, 99.7%) were acquired from Macklin Biochemical Co., Ltd. (Shanghai, China). Rhodamine B isothiocyanate (RBITC, C_29_H_30_ClN_3_O_3_S) was purchased from Yuanye Biotech Co., Ltd. (Shanghai, China). Cell culture reagents DMEM/F12 and DMEM were purchased from Gibco (Waltham, MA, USA). Aflibercept (Afl, 40 mg/mL) was purchased from Novartis (Basel, Switzerland). VEGF-A_165_ was from Med Chem Express LLC (Monmouth Junction, NJ, USA). Phosphate-Buffered Saline (PBS, pH 7.3 ± 0.1), 2-(4-Amidinophenyl)-6-indolecarbamidine dihydrochloride (DAPI, C_16_H_15_N_5_), and 1,1′-dioctadecyl-3,3,3′,3′-tetramethylindocarbocyanine perchlorate (DiI, C_59_H_97_ClN_2_O_4_) were from Beyotime Biotechnology Co., Ltd. (Shanghai, China). Fetal Bovine Serum were from Thermo Fisher Scientific Inc., (Waltham, MA, USA). Human umbilical vein endothelial cells (HUVEC) and human retinal epithelial cells (ARPE-19) lines were from Cell Culture Center, Institute of Basic Medical Sciences (Beijing, China).

### 2.2. Synthesis of S-MSN

The synthetic procedure was based on the previously reported methods to prepare S-MSN [41]. 0.1 g of hydroquinone, 0.14 mL of formaldehyde (37 wt%), and 3.0 mL of ammonia solution (28 wt%) were added to 10 mL of deionized water, and 70 mL of anhydrous ethanol in a triangular flask. The mixture was magnetically stirred at room temperature for 18 h. Then, 0.6 mL of TEOS was added, and the mixture was stirred at room temperature for 2 h. The products were collected by centrifugation. The reactants were alternately washed with ethanol and water three times. Then, the product was dried in a vacuum drying oven at 50 °C overnight. The next day, it was calcined at 550 °C for 5 h to obtain the S-MSN.

### 2.3. Synthesis of A-HDMSN

The synthetic procedure followed the previously reported methods to prepare A-HDMSN [37]. First, 0.3 g of TEA and 1 mL of CTAC solution were added to 9 mL of water, and the mixture was stirred at 60 °C for 1 h. Next, 9.5 mL of chlorobenzene and 25 µL of APTES were added to the mixed solution, then stirring was continued for 1 h at 60 °C. Then, 0.5 mL of TEOS was added to the mixture, and the reactants were stirred for 24 h. The sample was collected by centrifugation at 13,000 rpm for 10 min. The sediment was washed with ethanol. Finally, the surfactant was removed using hydrochloric acid and methanol solution. The solid product was dried in a vacuum desiccator to obtain A-HDMSN.

### 2.4. Synthesis of A-HDMSN-FITC

A 50 mmol/L FITC ethanol solution was prepared. 0.5 g of A-HDMSN and 20 μL of 50 mmol/L FITC solution were added to 1 mL of anhydrous ethanol solution. The mixture was stirred at 4 °C in the dark for 5 h. The reaction product was collected by centrifugation. Then, the reaction product was washed with ethanol. The reaction product was dried in a vacuum desiccator, and finally A-HDMSN conjugated with the FITC (A-HDMSN-FITC) were obtained. The chemical equation of the reaction was shown as follows in Equation (1):

R–N=C=S + SiO_2_–(CH_2_)_3_–NH_2_ → SiO_2_–(CH_2_)_3_-NH–CS–NH–R (1)

The fluorescence of A-HDMSN-FITC was examined using a handheld UV lamp (WFH-204B, Qiwei Instrument Co., LTD., Hangzhou, China) to verify characteristic yellowish-green emission. And the fluorescence spectrum of the A-HDMSN-FITC were determined by fluorescence spectra (Fluoromax-4, HORIBA, Ltd., Kyoto, Japan) with an excitation wavelength at 495 nm.

### 2.5. Instrumental Characterizations

The morphologies of S-MSN were observed using a scanning electron microscope (SEM, SU 8020, Hitachi, Tokyo, Japan) and transmission electron microscope (TEM, JEM-1400, JEOL, Tokyo, Japan). The structural and chemical properties of S-MSN were characterized by X-ray diffraction (XRD, X’Pert PRO, PANalytical, Almelo, The Netherlands) and Fourier-transform infrared spectroscopy (FTIR, Nicolet iS50 spectrometers, Thermo Fisher Scientific Inc., Waltham, MA, USA). Nitrogen adsorption–desorption isotherms were measured with a Micromeritics Tristar 3020 analyzer (Micromeritics, Norcross, GA, USA).

### 2.6. Drug Loading Capacity and Loading Efficiency of S-MSN and A-HDMSN

For the drug loading, a mixture of S-MSN and Afl at a ratio of 2:1 was prepared in 1 mL of PBS, and a mixture of A-HDMSN and Afl at a ratio of 2:1 was prepared in 1 mL of buffer. The suspension was stirred at 4 °C for 24 h. Afterwards, the mixture was centrifuged at 12,000 rpm for 5 min, and the supernatant was used to quantify the residual amount of drug by a Nanodrop (NanoDrop 2000C, Thermo Fisher Scientific, Waltham, MA, USA).

The drug loading capacity was calculated as Equation (2): *DL* is mean drug loading capacity; *C_I_* is mean initial drug concentration, µg/mL; *C_R_* is mean residual drug concentration, µg/mL; *V* is mean total volume, mL; *W_NP_* is mean weight of MSN nanoparticles (mg).

(2) $$DL\;(µg/mg)=\frac{\left(C_{I}-C_{R}\right)\times V}{W_{NP}}$$

The loading efficiency was calculated as Equation (3): *LE* is mean loading efficiency.

(3) $$LE\;(\%)=\frac{\left(C_{I}-C_{R}\right)\times V}{W_{NP}}\times 100\%$$

### 2.7. Release Curve of A-HDMSN

For the release of Afl, Afl was labeled with Rhodamine B isothiocyanate (RBITC). To make RBITC-Afl conjugates, Afl solution (10 mg/mL in PBS solution) was mixed with RBITC solution (250 mg/mL in DMSO solution) at a mass ratio of 1:1 (RBITC: Afl). Then, Afl concentration was adjusted to 1 μg/μL with PBS. The reaction system was shaken for 24 h at 4 °C under dark conditions. The chemical equation of the reaction was shown as follows in Equation (4):

R–N=C=S + R’–NH_2_ → R–NH–CS–NH–R’ (4)

The reaction mixture was subjected to dialysis under dark conditions. The 1 mg/mL A-HDMSN loaded with RBITC-labeled Afl were dispersed in PBS. At specific time points, the eluent was collected, and 1 mL of fresh PBS was added to the A-HDMSNs. The collected supernatant was analyzed to measure concentration of the released Afl using the fluorescent intensity at 553 nm.

We verified the successful conjugation of Afl and RBITC by gel electrophoresis. 10 μL of Afl-RBITC were mixed with loading buffer. Electrophoresis was operated at 60 V voltage for 2 h. Then, the gel was photographed under fluorescent (540 nm excitation, 610 nm emission) and bright field.

Similarly, we also verified the integrity of the Afl released from Afl (not conjugation with RBITC)-loaded A-HDMSN by gel electrophoresis. The release fluid of Afl-loaded A-HDMSN, as the sample, were mixed with loading buffer. The electrophoresis conditions remain unchanged. Stain the gel with Coomassie brilliant blue.

### 2.8. Cytotoxicity Assay

Cell viability in the presence of A-HDMSN was evaluated by using MTT assay. The ARPE-19 or HUVEC were seeded in 96-well plates at a density of 1.0 × 10^4^ cells per well and grown in an incubator at 37 °C and 5% CO_2_. After 24 h, the medium was removed, and 100 μL of fresh medium with different concentrations of A-HDMSN were added, followed by incubation for 48 h. Then, the cells were treated according to the protocol of MTT assay protocol [42], and the absorbance of each well at 490 nm was measured using a microplate reader (Infinite M200, Tecan, Zurich, Switzerland).

### 2.9. Cell Uptake of A-HDMSN

ARPE-19 cells were seeded in confocal microdishes (Nest, Wuxi, China) at a density of 1.0 × 10^5^ cells/well and cultured in DMEM/F12 medium. The next day, the culture medium was replaced by fresh medium containing 1 mg/mL A-HDMSN or A-HDMSN-FITC. The negative group continued to be cultured in the medium. The culture medium was discarded after 5 h, and the cells were washed three times with PBS. The cell membranes were stained with 2.5 μg/mL DiI in PBS for 15 min, and the cell nuclei were stained with 1 μg/mL DAPI in PBS for 5 min. After DiI and DAPI staining, the cells were washed three times with PBS. Then, cellular uptake images were taken by using laser scanning confocal microscopy (LSM880, Zeiss, Jene, Germany).

The uptake of free drugs by ARPE-19 cells was also investigated. The experimental process is similar with the previous one. Seed the cells in confocal microdishes. On the next day, co-culture the cells with medium, Afl, or Afl conjugated with RBITC. After 5 h, take photos with an inverted fluorescence microscope.

### 2.10. Flow Cytometry

ARPE-19 cells were seeded in 12-well plates at a density of 1.0 × 10^5^ cells per well. The cells were cultured overnight in DMEM/F12 medium. Next day, the culture medium was discarded and replaced with culture medium containing 1mg/mL A-HDMSN or A-HDMSN-FITC. After co-culture for 1, 3, 5, 7, 9, 12, and 24 h, the cells were washed 3 times with PBS. Cells were treated with trypsin digestion solution. Then, the trypsin was discarded, and the cells were resuspended and collected. The fluorescence intensity values of collected cells were detected by flow cytometry (FACS Calibur, Milpitas, CA, USA).

### 2.11. Cell Migration Assay

HUVEC were seeded in 24-well plates at 1 × 10^5^ cells per well and grown in an incubator at 37 °C and 5% CO_2_. Upon reaching 90% confluency, the cell monolayer was mechanically wounded using a sterile 200 μL yellow pipette tip. The cells were then washed 3 times with PBS to remove the detached cells and scratches were imaged by using an inverted microscope (IX71, Olympus, Hachioji, Japan). Subsequently, the cells were incubated with 2 mL culture medium without (negative group) or with 20 ng/mL VEGF-A_165_ (MCE LLC, Monmouth Junction, NJ, USA). To test the inhibitory effect of drug-loaded A-HDMSN on VEGF-A_165_, 1 mg/mL of untreated A-HDMSN@Afl or A-HDMSN@Afl incubated in the PBS for 10 days to release the drug early was simultaneously added with VEGF-A_165_. After incubation for 48 h, the scratches were imaged again using inverted microscope.

### 2.12. Statistical Analysis

All data were analyzed with SPSS 26.0 statistics software (SPSS 26.0, IBM Corporation, Armonk, NY, USA). Data are expressed as mean ± standard (SD) error of mean.

## 3. Results and Discussion

### 3.1. Preparation and Morphological Characterization of S-MSN and A-HDMSN

**Figure 1:**
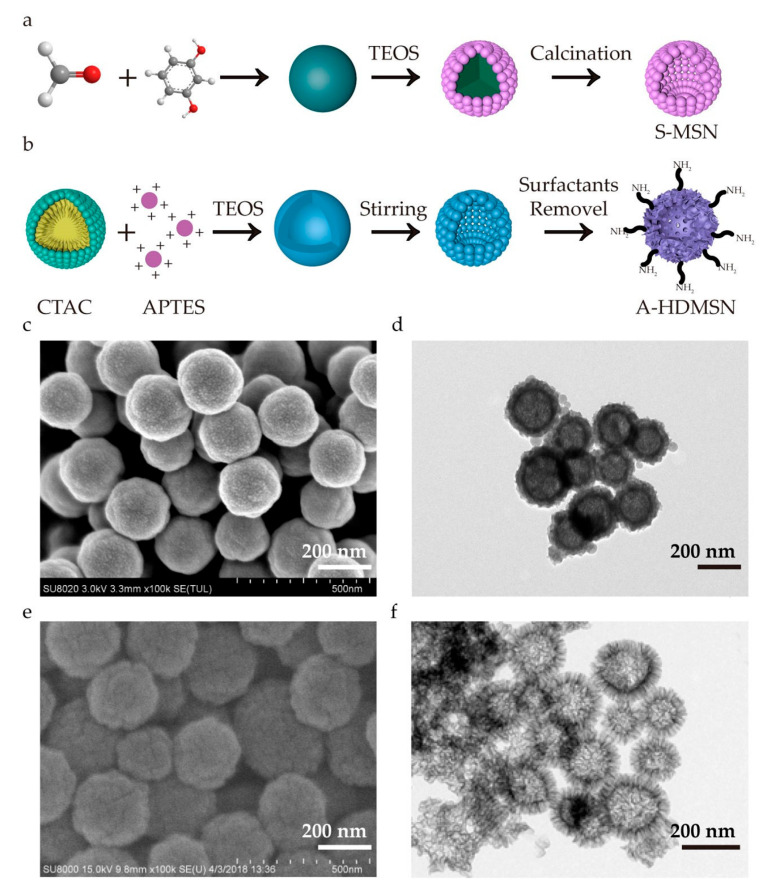
The prepared protocol and surface morphology of S-MSN and A-HDMSN. (**a**) Schematic diagram of the S-MSN synthesis mechanism. (**b**) Schematic diagram of the A-HDMSN synthesis mechanism. (**c**) The SEM of S-MSN. (**d**) The TEM of S-MSN. (**e**) The SEM of A-HDMSN. (**f**) The TEM of A-HDMSN. The scale is 200 nm.

Shell–nucleus structure MSNs were engineered through a multi-step templating approach to optimize drug loading capacity. The synthetic strategy and formation mechanism were systematically presented in Figure 1. A-HDMSN were synthesized using cetyltrimethylammonium chloride (CTAC) as the surfactant, triethylamine (TEA) as the catalyst, and TEOS and 3-aminopropyl triethoxysilane (APTES) as the silicon sources (Figure 1a). Formaldehyde reacted with resorcinol to obtain phenolic resin microspheres as the core of silica. Then, using TEOS as the silicon source, S-MSN with a core–shell structure were obtained after calcination and removing the phenolic resin microspheres (Figure 1b). The internal cavities of S-MSN and A-HDMSN served as storage chambers, which enhanced the storage of guest molecules. The SEM images (Figure 1c) and TEM images (Figure 1d) showed the well-dispersed S-MSN. The SEM images (Figure 1e) and TEM images (Figure 1f) showed the well-dispersed A-HDMSN. The diameters of these S-MSN were 179.61 ± 15.37 nm. The diameters of the A-HDMSN were 214.92 ± 32.81 nm with spike lengths of 50.23 ± 6.10 nm, which exhibited an inner hollow structure and obvious outer surface nanotopographies. The hollow structure provided the possibility for drug loading. Meanwhile, we illustrated the SEM images of S-MSN and A-HDMSN with low magnification in Figure S1a,b. The histogram of the size distribution are illustrated in Figure S1c,d. The hydrated particle sizes of S-MSN, A-HDMSN, and FITC-labeled A-HDMSN were 317.27 ± 9.85 nm (PDI = 0.19 ± 0.09), 369.65 ± 24.39 nm (PDI = 0.24 ± 0.02), and 370.33 ± 40.45 nm (PDI = 0.29 ± 0.03), respectively, as determined by dynamic light scattering (DLS). The zeta potential of S-MSN and A-HDMSN were -23.52 ± 0.40 mV and 9.1 ± 0.53 mV, respectively, indicating that the particle size of S-MSN and A-HDMSN were uniform.

### 3.2. XRD and FTIR Characterizations of S-MSN and A-HDMSN

**Figure 2:**
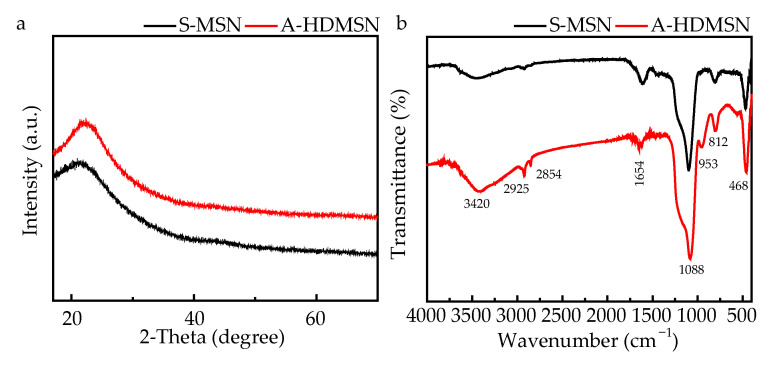
Characterization of S-MSN and A-HDMSN. (**a**) XRD patterns of S-MSN (black line) and A-HDMSN (red line). (**b**) FTIR pattern of S-MSN (black line) and A-HDMSN (red line).

To elucidate the structural and chemical characteristics of the synthesized materials, comprehensive XRD and FTIR analyses were performed on both S-MSN and A-HDMSN samples. The XRD patterns of S-MSN and A-HDMSN (Figure 2a) exhibited a characteristic broad peak centered at 2θ ≈ 22°, which was distinctive in amorphous silica structures, both in S-MSN and A-HDMSN [43]. This diffuse scattering profile, devoid of sharp Bragg reflections, confirmed the predominantly disordered atomic arrangements within the material. The FTIR spectroscopy (Figure 2b) revealed characteristic vibrational modes of mesoporous silica structures. In Figure 2b, the asymmetric stretching vibration absorption peaks belonging to silicon dioxide Si-O-Si at 1088 cm^−1^ and 953 cm^−1^ [44] are shown. The symmetric stretching vibration absorption peaks of silicon dioxide Si-O-Si were observed at 812 cm^−1^ and 468 cm^−1^ [45]. The absorption peaks at 2925 cm^−1^ and 2854 cm^−1^ belong to the asymmetric and symmetric -CH_2_-, which may result from the APTES and residual trace surfactant CTAC [46]. The characteristic peak of the bending vibration model corresponding to the O-H bond was observed at 1654 cm^−1^ [47]. The absorption peak at 3420 cm^−1^ was caused by the asymmetric stretching vibration resulting from the O-H bonds of the unremoved water molecules in the sample. The absorption peak at 3420 cm^−1^ (red line) may also originate from the stretching vibration of the N-H bond, which is different from S-MSN, which might be attributed to primary and secondary amines. The original individual spectra of FTIR are illustrated in the Figure S2. To sum up, these spectroscopic results confirm the amorphous silica framework in both materials and reveal the presence of surface functional groups (-NH_2_) that distinguish it from S-MSN.

### 3.3. Drug Loading of S-MSN and A-HDMSN

**Figure 3:**
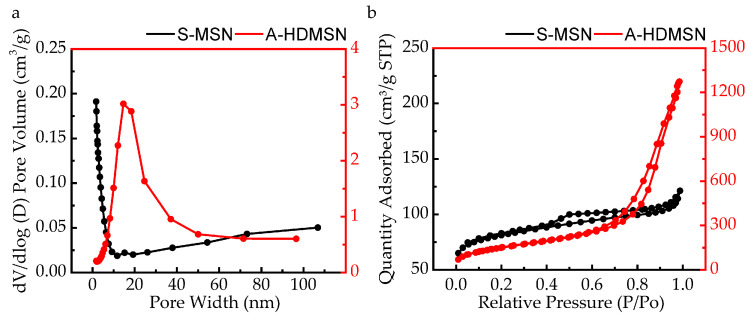
N_2_ adsorption and desorption experiment. (**a**) Nitrogen adsorption and desorption isotherms of S-MSN (black line) and A-HDMSN (red line). (**b**) BJH pore size distribution curves derived from adsorption branches of S-MSN (black line) and A-HDMSN (red line).

Considering the key requirements for Afl delivery carriers, we investigated whether the pore structures of S-MSN and A-HDMSN matched with Afl by N_2_-adsorption porosimetry [48]. To determine the BET processing temperature, we analyzed the sample stability using TG-DSC. The TG-DSC analysis results of S-MSN and A-HDMSN from the thermogravimetric analyzer are shown in Figure S3. During the temperature range from 0 °C to 150 °C, the weight loss of S-MSN and A-HDMSN were the same, both 0.50%. This weight loss is attributed to the removal of free and adsorbed water from the S-MSN and A-HDMSN as the temperature increases. This is the appropriate pretreatment temperature for BET. The BET test results are shown in Figure 3a. The pore size of S-MSN was mainly distributed below 10 nm. However, the size of Afl is approximately 10 nm (115 kDa), which was a mismatch for S-MSN loading. The pore size of A-HDMSN was centered at approximately 17 nm, and it exhibited a unimodal mesoporous structure, matching the requirements for Afl loading. Figure 3b shows obvious hysteresis loop in the nitrogen adsorption/desorption isotherms of A-HDMSN and S-MSN, which were identified as type IV nitrogen adsorption/desorption isotherm. The pore volume of A-HDMSN was 1.97 cm^3^/g, while that of S-MSN was 0.19 cm^3^/g. The specific surface area of A-HDMSN was 550.32 m^2^/g, which was significantly higher than that of S-MSN (257.72 m^2^/g). Due to the significant advantages in specific surface area and pore volume, A-HDMSN had a large drug loading capacity of 286.31 ± 8.14 μg/mg, and the loading efficiency was 28.63%. The drug loading of S-MSN was 54.26 ± 3.61 μg/mg, and the loading efficiency was 5.42%. There is an extremely significant difference between A-HDMSN and S-MSN. Based on these data, A-HDMSN were found to be more suitable for loading Afl as a vector. In subsequent experiments, we further verified the cellular uptake behavior of A-HDMSN and the therapeutic effect after drug loading.

### 3.4. Antibody Integrity and Release Behavior of A-HDMSN

The release study of anti-VEGF drug-loaded MSN was illustrated in Figure S3. We labeled Afl with RBITC and confirmed the firmness of the binding between RBITC and Afl by gel electrophoresis. A pink band appeared at 140 kDa in Figure 3a, slightly larger than 115 kDa (Afl). This is because RBITC conjugated to Afl increases its molecular weight. We excited RBITC-Afl with an excitation light of 540 nm and also observed fluorescence at 140 kDa (Figure S3b). It demonstrates the conjugation of RBITC and Afl. Subsequently, we quantified Afl using fluorescence intensity and plotted the release curve. The release period lasted for 19 days (Figure S3c). The release curve conforms to the Korsmeyer-Peppas model. To verify the integrity of Afl released from A-HDMSN, we conducted SDS-PAGE using the A-HDMSN@Afl (Afl is not connected to RBITC)-released extracellular fluid. The SDS-PAGE results show (Figure S3d) that Afl appears at a molecular weight of approximately 115 kDa. It indicates that the Afl released by A-HDMSN is still a complete antibody.

### 3.5. FITC Conjugated with A-HDMSN

**Figure 4:**
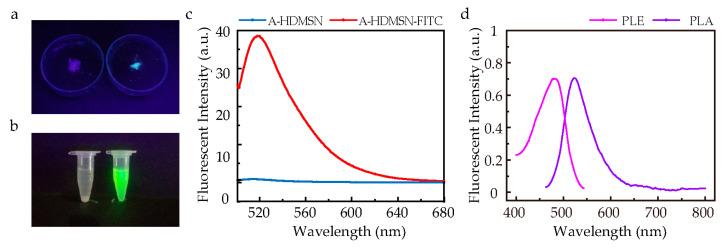
The samples were photographed under ultraviolet light and fluorescence spectrum. (**a**) Shows dried samples of A-HDMSN (left) and A-HDMSN-FITC (right). (**b**) Displays A-HDMSN nano-suspension (left) and A-HDMSN-FITC nano-suspension (right). (**c**) Fluorescence spectrum of A-HDMSN (blue line) and A-HDMSN-FITC (red line). (**d**) The PLA-PLE spectra of A-HDMSN-FITC.

In order to trace the cellular uptake behavior of A-HDMSN, it is necessary to construct A-HDMSN fluorescent probes. The surface amine groups of A-HDMSN were conjugated with FITC via thiourea bond formation, achieved by nucleophilic addition of the -NH_2_ group to the isothiocyanate’s C=S bond in FITC, yielding fluorescent A-HDMSN-FITC probes. The appearance of A-HDMSN (left) and A-HDMSN-FITC (right) after vacuum drying is shown in (Figure 4a), while Figure 4b shows photos of A-HDMSN (left) and A-HDMSN-FITC (right) nano-suspension. Under ultraviolet light irradiation, A-HDMSN-FITC exhibited a strong yellowish-green fluorescence, whereas A-HDMSN were non-fluorescent. These observations collectively demonstrated the conjugation of FITC to the amino-functionalized silica surface. To further validate the conjugation between FITC and A-HDMSN, fluorescence spectroscopy was employed to characterize the A-HDMSN-FITC. Figure 4c presents the fluorescent spectra of the A-HDMSN-FITC and A-HDMSN nano-suspensions. When excited at 495 nm, the fluorescence spectra showed a maximum emission for A-HDMSN-FITC at 520 nm, while no emission was detected of A-HDMSN. The spectroscopic results confirm the successful grafting of FITC onto the A-HDMSN and corroborate the visual observations presented in Figure 4a,b, demonstrating the formation of the fluorescent A-HDMSN-FITC conjugate. Luminescence spectra were also carried out as PLA-PLE spectra for greater clarity, as shown in Figure 4d. The maximum excitation wavelength was about 490, and the maximum emission wavelength is 520.

### 3.6. Cytotoxicity of A-HDMSN

**Figure 5:**
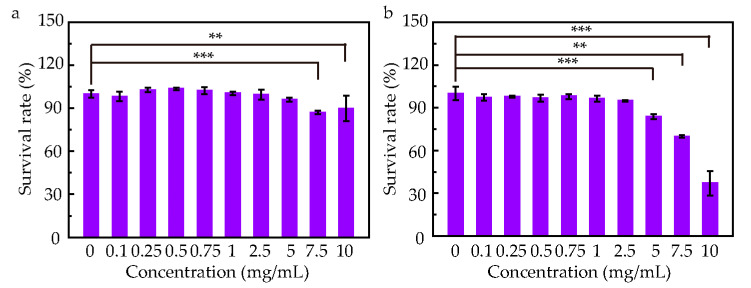
The safety assessment primarily evaluates the cytotoxicity of the synthesized A-HDMSN toward distinct cell lines. (**a**) The survival rate of ARPE-19 after co-cultured with 0.1, 0.25, 0.75, 1, 2.5, 5, 7.5, and 10 mg/mL A-HDMSN for 48 h. (**b**) The survival rate of HUVEC after co-cultured with 0.1, 0.25, 0.75, 1, 2.5, 5, 7.5, and 10 mg/mL A-HDMSN for 48 h. (** *p* < 0.01, *** *p* < 0.001).

To ensure ocular safety, we selected ARPE-19 and HUVEC because the former represents the outer blood–retinal barrier and the latter mirror the retinal vasculature—both critical tissues that an intraocular nanocarrier will encounter. To evaluate the biocompatibility of A-HDMSN, we performed MTT cytotoxicity assays on these cells. As shown in Figure 5a, the cell viability remained above 85% at 0.1–10 mg/mL for ARPE-19 when compared with untreated group (0 dose group), indicating negligible toxicity. Meanwhile, in HUVEC, it reveals that cell viability exceeded 94% at 0.1–2.5 mg/mL, decreased to 84% at 5 mg/mL, and dropped to 37% at 10 mg/mL (Figure 5b). These results demonstrate that A-HDMSN is cytocompatible at concentrations up to 7.5 mg/mL. The toxicity of Afl to ARPE-19 and HUVEC was also investigated. Figure S5a,b illustrate the safety of Afl co-incubation with ARPE-19 and HUVEC at 0.1–10 mg/mL, respectively. Figure S5c,d illustrates the safety of Afl-loaded A-HDMSN co-incubation with ARPE-19 and HUVEC. There was no statistically significant difference between the Afl-loaded A-HDMSN and the non-administration group for ARPE-19, at 0.1 to 5 mg/mL. And no statistically significant difference between the Afl-loaded A-HDMSN and the non-administration group for HUVEC, at 0.1 to 1 mg/mL.

### 3.7. ARPE-19 Uptake of A-HDMSN

**Figure 6:**
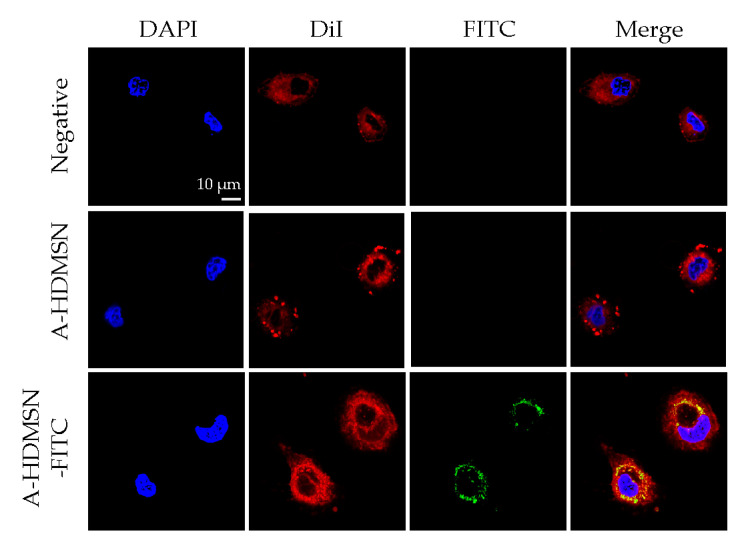
ARPE-19 cell uptake for culture medium (negative group), A-HDMSN, and A-HDMSN-FITC. The scale is 10 μm.

To investigate the cellular uptake behavior of MSN, we incubated ARPE-19 cells with FITC-labeled A-HDMSN for 4 h and imaged them by confocal microscopy. Results showed that negative controls and unlabeled A-HDMSN displayed only blue nuclear and red membrane staining without green fluorescence, whereas the A-HDMSN-FITC group exhibited strong green fluorescence in the cytoplasm (Figure 6), indicating efficient endocytosis and cytoplasmic localization of the carrier by ARPE-19 cells. Meanwhile, we investigated the uptake results of free drugs by ARPE-19 cells. The Afl can hardly be taken up by APRE-19 cells (Figure S6).

**Figure 7:**
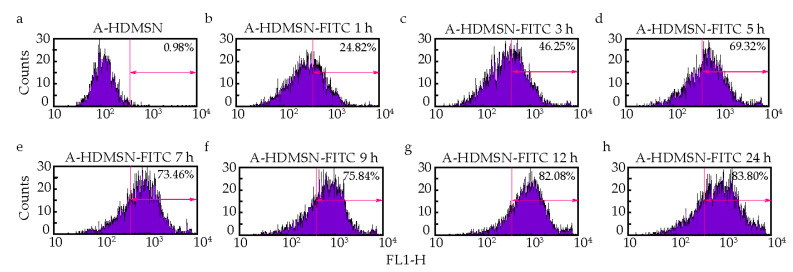
Quantitative analysis ARPE-19 cell uptake for A-HDMSN-FITC. (**a**) A-HDMSN group, A-HDMSN was co-cultured with ARPE-19. (**b**–**h**) A-HDMSN-FITC group, A-HDMSN-FITC was co-cultured with ARPE-19 for 1, 3, 5, 7, 9, 12, and 24 h.

To further quantitatively analyze whether cell uptake behavior is related to incubation time, we detected the fluorescence intensity of A-HDMSN-FITC co-incubated with ARPE-19 cells for 1, 3, 5, 7, 9, 12, and 24 h using flow cytometry. Results showed that the percentage of positively stained cells increased over time. Specifically, the proportion of positive cells was 24.82% at 1 h, rising significantly to 69.32% by 5 h. The uptake continued to increase, reaching 83.80% at 24 h (Figure 7). This trend indicates that the cellular uptake of A-HDMSN-FITC is time-dependent, with the highest uptake observed at the 24 h mark, demonstrating saturation of the cellular uptake process.

### 3.8. VEGF Inhibitory Effect of A-HDMSN@Afl

**Figure 8:**
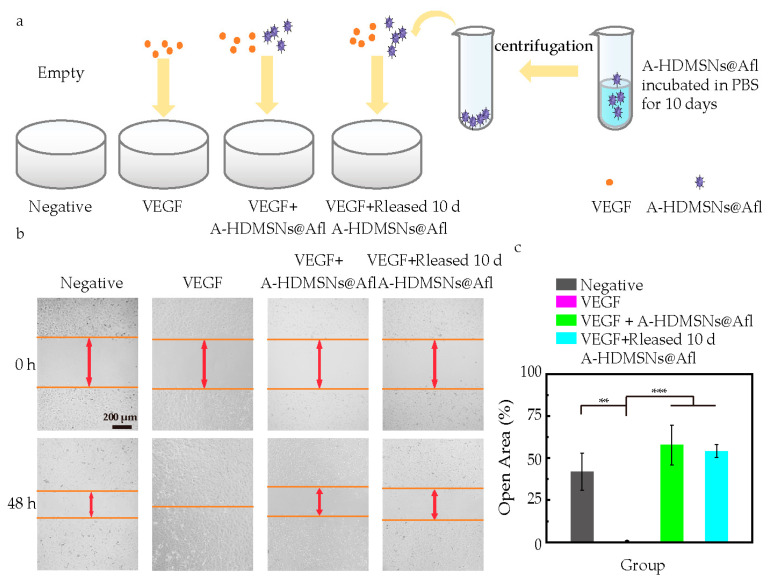
Afl-loaded A-HDMSN inhibit VEGF-induced cell migration. (**a**) Grouping situation of scratch tests. (**b**) The image of scratch wound migration assay. (**c**) The open area% of the cell scratch were put through quantification analysis. (n = 6) (** *p* < 0.01, *** *p* < 0.001). The scale is 200 μm.

The aforementioned cytotoxicity and cellular uptake results demonstrate that the A-HDMSN we have prepared exhibit favorable biocompatibility and the capability to be internalized by retinal cells. Therefore, in the next step, we loaded A-HDMSN with Afl (A-HDMSN@Afl) and verified the bioactivity of the Afl loaded in A-HDMSN. We assessed the VEGF inhibitory effect of A-HDMSN@Afl to determine its potential in controlling HUVEC cell migration. Grouping situation of scratch tests is shown in Figure 8a. As shown in Figure 8b, the results indicated that the migration of HUVEC in the VEGF-_165_ group was significantly faster than that in the control group, due to the proliferation and migratory effects of VEGF-_165_ [49]. Almost 100% of the open areas in the VEGF group were covered by migratory HUVEC. However, co-administration of A-HDMSN@Afl with VEGF-_165_ treatment led to a marked attenuation of VEGF-_165_-induced migratory enhancement in HUVEC (Figure 8b), which is also reflected in the scratch open area, almost the same as the control group (Figure 8c) These observations indicate that A-HDMSN@Afl exerts a potent inhibitory effect on VEGF-_165_ activity throughout the 48 h duration of the cell migration assay.

Furthermore, to assess a longer time-sustained release capability of A-HDMSN, we pre-incubated A-HDMSN@Afl with the PBS for 10 days to allow for drug release before exposing it to the cells. As depicted in Figure 8c, at the 48 h time point, the open area percentages of the A-HDMSN@Afl and the 10-day released A-HDMSN@Afl groups were 57.88 ± 11.90% and 54.16% ± 3.85%, respectively. In contrast, the VEGF group displayed a significantly lower normalized open area of 0.3 ± 0.37%. All data were normalized to the initial values at 0 h via ImageJ software (V1.45 NIH, Bethesda, MD, USA) for quantitative analysis. This suggests that A-HDMSN can effectively extend the release of Afl for at least 10 days, compared with the half-life of Afl in vitreous body (only 3 days) [20], highlighting its potential as a long-acting anti-VEGF agent. It shows the potential to prolong the clinical administration time for patients, which can not only reduce the suffering of patients but also alleviate the financial burden.

## 4. Conclusions

We prepared two kinds of silica nanoparticles, S-MSN and A-HDMSN. The A-HDMSN has a larger pore size of 17 nm; however, the pore size of S-MSN is only below 10 nm. The particle size of the Afl was 10 nm. Therefore, the pore size of S-MSN does not match that of Afl, preventing Afl from entering the interior of S-MSN. In contrast, the pore size of A-HDMSN matches that of Afl, allowing Afl to enter the interior of A-HDMSN and thereby enhancing the drug loading capacity. Our experimental results were consistent with these. A-HDMSN exhibited a strong drug loading capacity of 286.31 μg/mg, whereas S-MSN had a capacity of 54.26 μg/mg for Afl.

**Figure 9:**
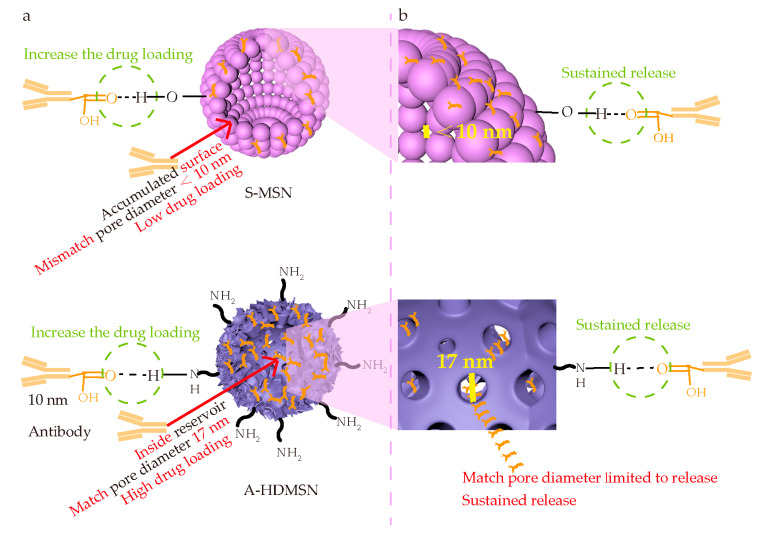
Schematic illustration of drug loading and release mechanisms. (**a**) Schematic diagram of S-MSN and A-HDMSN-loaded Afl. (**b**) Schematic diagram of S-MSN and A-HDMSN-released Afl.

We have analyzed the reasons for the loading and release of drugs as follows. In terms of drug loading, the pore structure of A-HDMSN is 17 nm, allowing 10 nm of antibodies to enter its storage chamber. It has a relatively large drug loading capacity. However, the pore size of S-MSN is below 10 nm, which restricts Afl entering its storage chamber, causing Afl to mainly adsorb on the surface of S-MSN. The drug loading capacity was substantially reduced. Furthermore, the specific surface area of A-HDMSN is 2.14 times that of S-MSN, which increases its drug loading capacity. Meanwhile, both S-MSN and A-HDMSN can form hydrogen bonds with Afl, which is of positive significance for increasing the drug loading capacity. Overall, the drug loading capacity of A-HDMSN is 5.28 times that of S-MSN, which is mainly due to its hydrogen bonding ability and matched pore size to Afl [50]. In terms of drug release, as shown in Figure 9b, the release of Afl-loaded S-MSN only depends on the hydrogen bond. In contrast, the sustained release of Afl-loaded A-HDMSN may be due to the combined action of two aspects. First, it may be due to the hydrogen bonding between Afl and A-HDMSN. Secondly, the confined pore size of A-HDMSN (17 nm) imposes diffusion constraints of Afl (10 nm), enabling the sustained and controlled diffusion of Afl from the storage chamber. This interaction is essential for Afl delivery without diminishing the affinity for VEGF. Hydrogen bonds and pore channel constraints synergistically increase the release time of A-HDMSN@Afl. Based on the above, a possible high loading capacity and long-term released mechanism was proposed for A-HDMSN and S-MSN, as shown in Figure 9a.

A-HDMSN could be modified with fluorescent groups and tracked within cells. We demonstrated that the uptake behavior of A-HDMSN-FITC by cells was positively correlated with the co-incubation time within 1–12 h. Both the A-HDMSN@Afl group and A-HDMSN@Afl-released 10 days group inhibited VEGF-induced cell migration. This indicates that A-HDMSN could release Afl in at least 10 days, providing long-term anti-VEGF treatment. This work demonstrates that through surface modification and pore size regulation, we have synthesized A-HDMSN as carriers to match Afl. The A-HDMSN are highly promising for prolonging the intravitreal administration time in AMD treatment, expanding the application of silica NPs in ophthalmology and providing valuable insights for the modification and enhancement of traditional ocular medications.

## Data Availability

The original contributions presented in this study are included in the article/Supplementary Material. Further inquiries can be directed to the corresponding authors.

## Supplementary Materials

The following supporting information can be downloaded at: https://www.mdpi.com/article/10.3390/ma18184384/s1, Figure S1. (a) SEM of S-MSN under a low-power. (b) SEM of A-HDMSN under a low-power. (c) Particle size distribution curves of S-MSN. (d) Particle size distribution curves of A-HDMSN. Figure S2. FTIR patterns of S-MSN and A-HDMSN. (a) FTIR patterns of S-MSN. (b) FTIR patterns of A-HDMSN. Figure S3. TG/DSC curves. (a) TG/DSC of S-MSN. (b) TG/DSC of A-HDMSN. Figure S4. (a) The gel photos of RBITC-Afl in bright field. (b) The gel fluorescent photos of RBITC-Afl. (c) Cumulative release curve. (d) Afl and released Afl from external liquid gel elec-trophoresis image stained with Coomassie brilliant blue. Figure S5. The safety assessment primarily evaluates the cytotoxicity of the Afl and A-HDMSN loaded Afl toward distinct cell lines. (a) The survival rate of ARPE-19 after co-cultured with 0.1, 0.25, 0.75, 1, 2.5, 5, 7.5 and 10 mg/mL Afl for 48 h. (b) The survival rate of HUVEC after co-cultured with 0.1, 0.25, 0.75, 1, 2.5, 5, 7.5 and 10 mg/mL Afl for 48 h. (c) The survival rate of ARPE-19 after co-cultured with 0.1, 0.25, 0.75, 1, 2.5, 5, 7.5 and 10 mg/mL A-HDMSN loaded Afl for 48 h. (d) The survival rate of HUVEC after co-cultured with 0.1, 0.25, 0.75, 1, 2.5, 5, 7.5 and 10 mg/mL A-HDMSN loaded Afl for 48 h. (* *p* < 0.05, ** *p* < 0.01, *** *p* < 0.001). Figure S6. ARPE-19 cell uptake for culture medium (negative group), Afl and Afl-RBITC. The scale is 10 μm.
