# Supplementary material for: Surface Modification and Pore Size Regulation of MSN as Function Aflibercept Carrier for Anti-Vascular Migration
Source: Materials (Basel). 2025 Sep 19;18(18):4384. doi: 10.3390/ma18184384 (PMC12471984; doi:10.3390/ma18184384)
Supplement: Supplementary file 1 [file materials-18-04384-s001.zip › materials-3782905-supplementary.pdf]

materials

MDPI

Supporting information

Surface Modification and Pore Size Regulation of MSN as Function Aflibercept Carrier for Anti-vascular migration

Ruiqi Guo [1], Xue Zhang [1], Yakai Song [1], Jiachen Shen [2], Kai Li [1*] and Yi Zheng [2*]

[1] Faculty of Life Science and Medicine, Harbin Institute of Technology, Harbin, 150080, China
[2] Eye Hospital, The First Affiliated Hospital, Harbin Medical University, Harbin, 150001, China
*Correspondence: zhengyi_edu@163.com (Zheng Y), likai2017@hit.edu.cn (Li K)

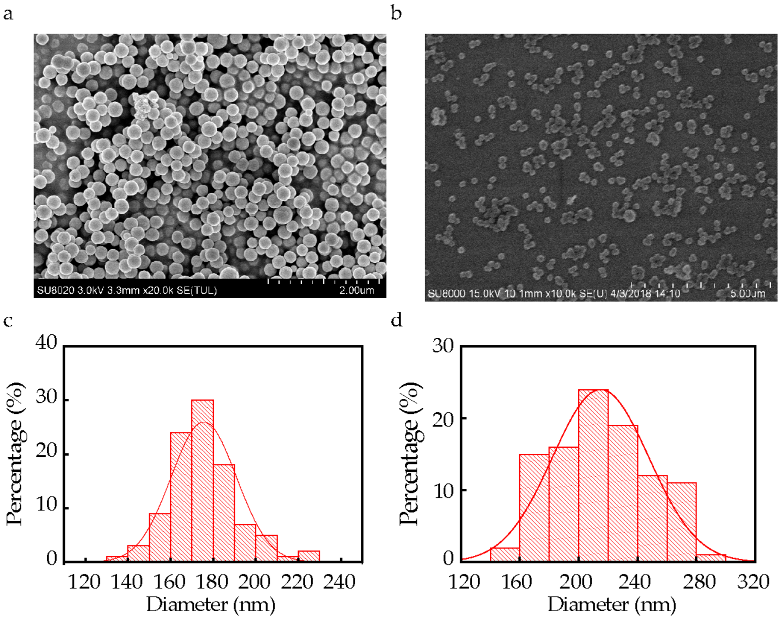

Figure S1. (a) SEM of S-MSN under a low-power. (b) SEM of A-HDMSN under a low-power. (c) Particle size distribution curves of S-MSN. (d) Particle size distribution curves of A-HDMSN.

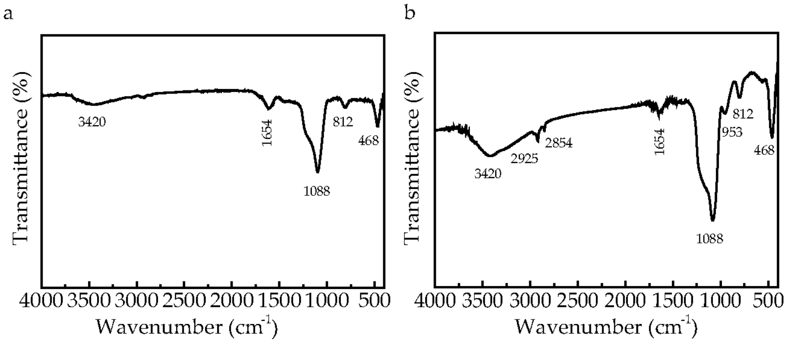

Figure S2. FTIR patterns of S-MSN and A-HDMSN. (a) FTIR patterns of S-MSN. (b) FTIR patterns of A-HDMSN.

 https://doi.org/10.3390/ma18184384

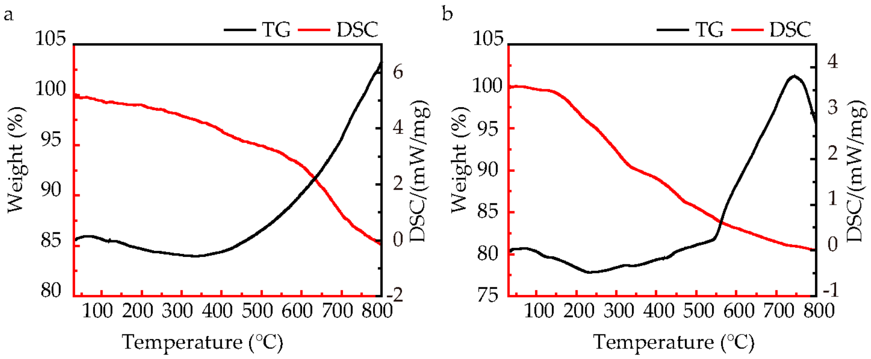

Figure S3. TG/DSC curves. (a) TG/DSC of S-MSN. (b) TG/DSC of A-HDMSN.

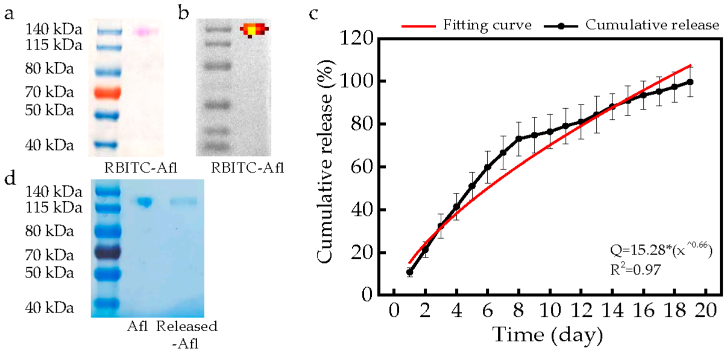

Figure S4. (a) The gel photos of RBITC-Afl in bright field. (b) The gel fluorescent photos of RBITC-Afl. (c) Cumulative release curve. (d) Afl and released Afl from external liquid gel electrophoresis image stained with Coomassie brilliant blue.

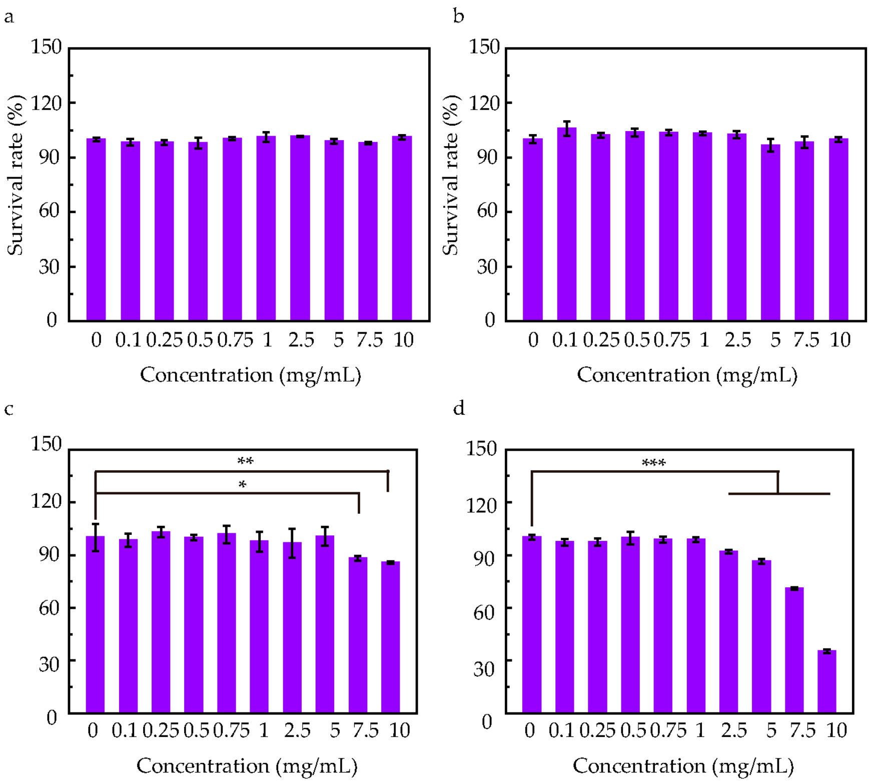

Figure S5. The safety assessment primarily evaluates the cytotoxicity of the Afl and A-HDMSN loaded Afl toward distinct cell lines. (a) The survival rate of ARPE-19 after co-cultured with 0.1, 0.25, 0.75, 1, 2.5, 5, 7.5 and 10 mg/mL Afl for 48 h. (b) The survival rate of HUVEC after co-cultured with 0.1, 0.25, 0.75, 1, 2.5, 5, 7.5 and 10 mg/mL Afl for 48 h. (c) The survival rate of ARPE-19 after co-cultured with 0.1, 0.25, 0.75, 1, 2.5, 5, 7.5 and 10 mg/mL A-HDMSN loaded Afl for 48 h. (d) The survival rate of HUVEC after co-cultured with 0.1, 0.25, 0.75, 1, 2.5, 5, 7.5 and 10 mg/mL A-HDMSN loaded Afl for 48 h. (*P < 0.05, **P < 0.01, ***P < 0.001).

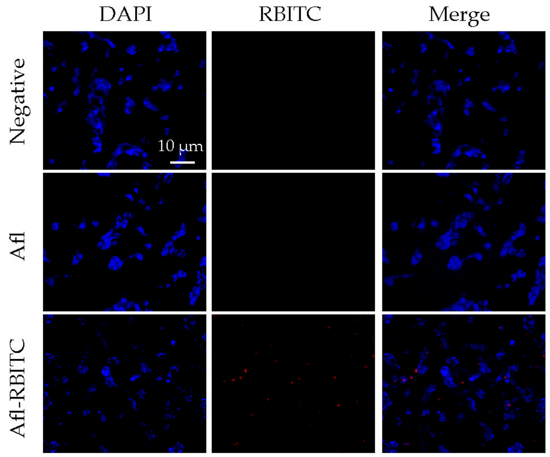

Figure S6. ARPE-19 cell uptake for culture medium (negative group), Afl and Afl-RBITC. The scale is 10 μm.
